# COP9 Signalosome’s Role in Plant Defense Mechanisms

**DOI:** 10.3390/plants14193017

**Published:** 2025-09-29

**Authors:** Zihua Lu, Chao Li, Kelin Deng, Cong Han, Zhihui Shan, Shuilian Chen, Hongli Yang, Yuanxiao Yang, Haifeng Chen, Qingnan Hao

**Affiliations:** 1 Oil Crops Research Institute, Chinese Academy of Agricultural Sciences, Wuhan 430062, China; 82101232218@caas.cn (Z.L.); lichao06@caas.cn (C.L.); 18369696664@163.com (K.D.); 821012450231@caas.cn (C.H.); shanzhihui@caas.cn (Z.S.); chenshuilian@caas.cn (S.C.); yanghongli@caas.cn (H.Y.); 15527183875@163.com (Y.Y.); 2 Graduate School, Chinese Academy of Agricultural Sciences, Beijing 100081, China; 3 Key Laboratory of Biology and Genetics Improvement of Oil Crops, Ministry of Agriculture and Rural Affairs, Wuhan 430062, China

**Keywords:** COP9 signalosome, plant disease resistance, plant–pathogen interaction, plant immunity

## Abstract

The COP9 signalosome (CSN) is a highly conserved eukaryotic protein complex that plays a crucial role in plant growth, development, and stress responses by modulating the ubiquitination pathway. Emerging evidence underscores its significance in plant immunity, where it orchestrates diverse defense mechanisms, including hormone signaling, reactive oxygen species (ROS), homeostasis, and secondary metabolite (SM) biosynthesis. As a key regulator, CSN influences multiple layers of immune responses, such as pattern-triggered immunity (PTI), effector-triggered immunity (ETI), and systemic acquired resistance (SAR). However, the intricate interplay between CSN and immune regulatory networks remains incompletely understood, and a comprehensive model of its mechanistic framework is still lacking. This review systematically consolidates current knowledge on CSN-mediated immune regulation in plant–pathogen interactions and highlights its role in disease resistance.

## 1. Introduction

Plants carry powerful and intricate defense mechanisms to counteract infection by pathogens. Plants employ a two-tiered immune system to counteract microbial invasion. The first line of defense, known as PTI, is initiated when cell surface-localized pattern recognition receptors (PRRs) detect conserved pathogen-associated molecular patterns (PAMPs) such as flagellin, chitin, lipopolysaccharides, and peptidoglycans [1,2]. This recognition triggers multiple defense responses, including callose deposition, ROS bursts [3], activation of mitogen-activated protein kinase (MAPK) cascades [4,5], and calcium ion flux modulation [6,7], collectively establishing robust basal immunity [8]. To overcome PTI, pathogens have evolved effector proteins that suppress these defense responses. In response, plants have developed a second layer of defense called ETI, mediated primarily by resistance (R) genes encoding nucleotide-binding leucine-rich repeat (NLR) proteins [9]. This process is known as the “Zig-Zag” model, which explains the ongoing battle between plants and pathogenic microbes, involving the activation and suppression of PTI and ETI [10]. ETI typically elicits stronger immune responses, often accompanied by programmed cell death (PCD) at infection sites [11,12]. Beyond sharing some components with PTI (such as ROS production and MAPK activation), ETI involves additional sophisticated defense mechanisms [1,13]. These include complex phytohormone signaling networks involving jasmonate (JA), salicylic acid (SA), ethylene (ET), and abscisic acid (ABA) [14]; enhanced biosynthesis of antimicrobial secondary metabolites; and significant upregulation of pathogenesis-related (PR) protein expression [8,15].

The COP9 signalosome is a highly conserved eukaryotic protein complex that plays pivotal roles in cellular regulation. In plants, the COP9 signalosome consists of nine subunits, with its structural core formed by CSN5 and CSN6, both harboring the Mpr1-Pad1 N-terminal (MPN) domain. The remaining six subunits (CSN1–CSN4, CSN7, and CSN8), which contain proteasome-COP9-eIF3 (PCI) domains, serve as the scaffolding framework of the complex [16]. Recent studies have further expanded the understanding of COP9’s composition by identifying COP9 signalosome-associated acidic protein (CSNAP) as an additional functional component [17].

Originally characterized in *Arabidopsis thaliana* as a key regulator of photomorphogenesis [18,19], the COP9 signalosome has since been implicated in diverse aspects of plant biology, including growth regulation, developmental processes, and stress responses [20]. A major focus of COP9 research centers on its critical role in modulating the ubiquitination pathway. Structural analyses revealed that CSN shares remarkable similarity with the lid complex of the 26S proteasome, through which it inhibits proteolytic activity. This inhibition is mediated by CSN’s ability to regulate the deneddylation of cullin subunits in RING-type E3 ubiquitin ligases, thereby exerting broad control over ubiquitin-dependent protein degradation. Through this pathway, CSN controls diverse processes, including hypocotyl elongation (via KELCH1) [21], floral organ development (mediated by unusual flower organs (UFOs)) [22], JA signaling (through coronatine-insensitive 1 (COI1)) [23], auxin response (via transport inhibitor response 1 (TIR1)) [24], gibberellin signaling (dependent on sleepy 1 (SLY1)) [25], and SA response (regulated by nonexpressor of PR genes 1 (NPR1)) [26]. The essential role of CSN is further evidenced by the severe phenotypes of cop9 loss-of-function mutants, which display stunted vegetative growth and complete reproductive failure, including impaired flowering and seed setting [27]. These findings collectively highlight the COP9 signalosome as a central regulatory hub that integrates multiple signaling pathways to coordinate plant growth, development, and environmental adaptation.

CSN has emerged as a pivotal regulator of plant immune responses against pathogen invasion. Accumulating evidence indicates that both the intact COP9 complex and its individual subunits play significant roles in modulating plant resistance to a broad spectrum of pathogens. Notably, transgenic plants with either overexpression or loss-of-function mutations in CSN subunits exhibit altered susceptibility to multiple phytopathogens, including *Magnaporthe oryzae* (rice blast), *Puccinia triticina* (wheat leaf rust), and *Erysiphe necator* (grapevine powdery mildew) [28,29]. This review aims to provide a comprehensive overview of the multifaceted roles of COP9 in plant defense mechanisms, encompassing ROS bursts, phytohormone signaling, secondary metabolite biosynthesis, and *R* gene-mediated immunity. Through this systematic exploration, we endeavor to clarify the mechanistic contributions of COP9 to plant disease resistance and its potential implications for crop improvement.

## 2. Structure and Function of COP9

**Figure 1:**
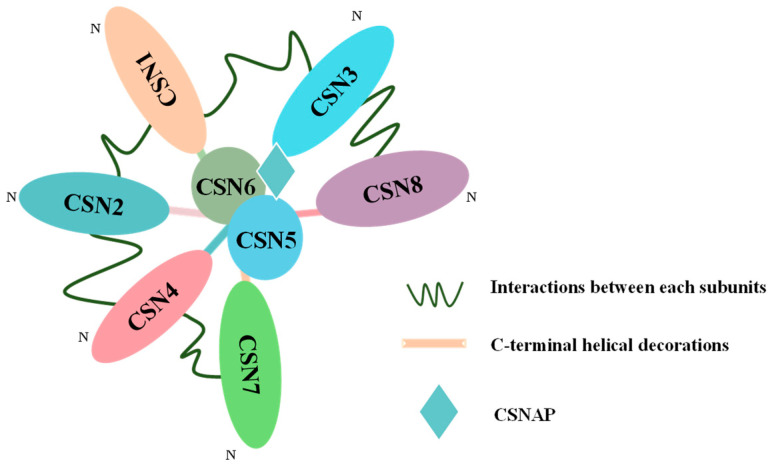
The structure of CSN. CSN is composed of nine subunits: CSN1–CSN8 and CSNAP. CSN1–CSN4 together with CSN7 and CSN8 interconnect to form a horseshoe-shaped open ring. The C-terminal PCI ring formed by six subunits joins CSN5 and CSN6, while CSNAP bridges CSN5, CSN6, and CSN3 to stabilize the complex. The diagram is a two-dimensional schematic with no specific scale.

The COP9 signalosome demonstrates striking structural conservation across eukaryotes, retaining a nearly identical architecture in humans, animals, and plants throughout evolution [30]. The complex adopts a “tomato-on-a-palm” configuration, consisting of nine subunits: CSN1–CSN8 and the recently characterized CSNAP [31]. The core of the complex is formed by CSN5 and CSN6, while CSN1–CSN4, CSN7, and CSN8 assemble into a peripheral ring (Figure 1). Structurally, six subunits harbor a PCI domain, a helical-rich motif critical for inter-subunit interactions and complex assembly [32]. The enzymatic core of CSN is defined by CSN5 and CSN6, which contain MPN domains. Notably, CSN5 possesses a catalytically active JAB1 MPN metalloenzyme (JAMM) domain, which coordinates a zinc ion to mediate isopeptidase activity, underscoring its central role in the complex [33]. In contrast, CSN6 lacks the metalloprotease motif and is thought to primarily stabilize the complex’s structural integrity [34].

A key advancement in CSN biology was the identification of CSNAP as an integral subunit through liquid chromatography–mass spectrometry (LC-MS) [17,35]. CSNAP interacts with the complex via its C-terminal F- and D-rich domain, specifically binding with CSN3, CSN5, and CSN6 (Figure 1). Structural analyses suggest that the COP9 complex exhibits considerable conformational flexibility, likely underpinning its functional plasticity [36]. Intriguingly, the stoichiometric balance between CSN5 and CSN6 is critical for regulating cullin deneddylation activity [37]. Further genetic studies in *Arabidopsis* highlight functional divergence between the paralogs CSN5A and CSN5B, revealing subunit-specific regulatory mechanisms in plant development and stress responses [38].

Cullin-RING E3 ubiquitin ligases (CRLs) constitute one of the largest ubiquitin ligase families, encompassing more than 200 complexes [39]. Each complex is built on a cullin scaffold that associates with a specific substrate receptor, an adaptor protein, and a RING protein that recruits the E2 enzyme [40,41]. Conjugation of the ubiquitin-like protein Nedd8 to the cullin subunit activates the CRL’s degradative capacity. CSN suppresses CRL E3 ubiquitin ligase activity by promoting the cleavage of the NEDD8–CUL1 conjugate, which is called deneddylation [42,43]. Upon CRL–CSN engagement, the CRLs dock between CSN2 and CSN4, weakening the CSN4–CSN6 interface and releasing the CSN5–CSN6 MPN dimer. The liberated CSN5 then binds the cullin scaffold and executes deneddylation [31,44].

Interestingly, emerging evidence suggests that CSN may also interact directly with the 26S proteasome [45,46]. The 26S proteasome consists of two key components: the 20S catalytic core and the 19S regulatory particle. The latter, often referred to as the “lid” complex, recognizes ubiquitinated substrates and facilitates their degradation. Striking structural homology has been observed between CSN and the 19S lid subcomplex, leading to the hypothesis that CSN may compete with or substitute the lid complex, thereby impairing proteasomal recognition and degradation of ubiquitinated proteins [47]. This model is supported by the accumulation of ubiquitinated proteins observed in CSN-deficient mutants [48]. Beyond its canonical roles in ubiquitination and proteasomal regulation, CSN has been implicated in diverse cellular processes. Multiple studies have demonstrated that CSN participates in transcriptional regulation and possesses intrinsic kinase activity [49,50,51]. These findings underscore the multifaceted nature of CSN as a pleiotropic regulatory complex in eukaryotic cells.

## 3. Various Pathogenic Microorganisms Target COP9

**Figure 2:**
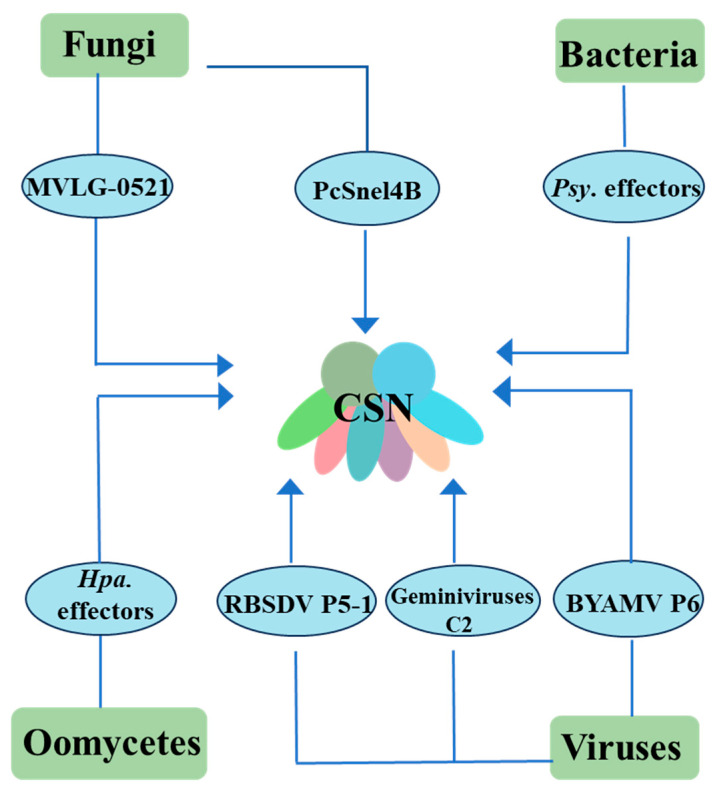
Multiple pathogens targeting CSN. This figure compiles currently known effector proteins that interact with CSN, originating from pathogens including fungi, bacteria, oomycetes, and viruses. (The arrows indicate the interactions.).

The co-evolutionary arms race between plants and pathogens has driven the development of increasingly sophisticated attack and defense strategies, as conceptualized in the “Zig-Zag” immunity model [9,10]. In this perpetual molecular conflict, pathogens have evolved an arsenal of virulence effectors capable of targeting host cellular processes. Notably, emerging research reveals that many pathogen effectors specifically target CSN as part of their infection strategy (Figure 2) [50,51,52,53,54]. This direct manipulation of the host’s ubiquitin-proteasome (UPS) machinery may represent an evolutionarily refined tactic to suppress plant immunity at its core.

For example, the fungal effector MVLG_05122 from *Microbotryum lychnidis-dioicae* has been shown to physically interact with the COP9 signalosome subunit OsCSN5a/b in rice. Notably, this interaction is localized at the base of leaf trichomes, implying that MVLG_05122 may hijack OsCSN5a/b to modulate trichome development, thereby facilitating pathogen infection [52]. Similarly, the *Phytophthora capsici* effector PcSnel4B suppresses cell death triggered by the resistance protein AtRPS2. Mechanistically, PcSnel4B directly binds to NbCSN5, interfering with its association with NbCUL1 and ultimately promoting the degradation of AtRPS2 [53]. These examples not only illustrate how pathogens manipulate the COP9 signalosome to their advantage but also underscore its role in regulating ETI.

Several plant viruses have evolved mechanisms to disrupt CSN, particularly through its fifth subunit, CSN5, to manipulate JA signaling. For instance, the rice black-streaked dwarf virus (RBSDV) encodes the P5-1 protein, which inhibits OsCSN5 function, leading to dysregulation of the JA pathway in rice [54]. Similarly, geminiviruses employ their C2 protein to interfere with CSN activity, impairing downstream JA signaling mediated by SCF-type E3 ubiquitin ligases [55]. Similarly, the insect-transmitted rhabdovirus barley yellow striate mosaic virus (BYSMV) exploits its accessory protein, P6, to target host CSN5. This interaction suppresses JA signaling, enhancing plant attractiveness to insect vectors and thereby facilitating viral transmission [56]. Notably, bacterial and oomycete pathogens also subvert CSN function. A total of 29 effector proteins from *Pseudomonas syringae* (*Psy*) and the obligate biotroph *Hyaloperonospora arabidopsidis* (*Hpa*) have been identified as CSN interactors. Consistent with this, *Arabidopsis thaliana* CSN5A mutants exhibit heightened resistance to both *Hpa.* and *Psy.*, underscoring the central role of CSN5 in plant immunity [57].

CSN has emerged as a common target for diverse pathogens, ranging from fungi, viruses, and bacteria to oomycetes, with its core subunit CSN5 being particularly vulnerable to pathogen manipulation. Over hundreds of millions of years of coevolution with plants, pathogens have evolved sophisticated infection mechanisms, yet they remain fixated on this highly conserved complex. What role does COP9 play in plant immunity to make assailants so “persistent”? On one hand, due to the high conservation of COP9 across species, it also regulates the growth and development of pathogenic microorganisms [58,59,60]. The translation and homeostasis of microbial effector proteins may be linked to COP9, and the binding of some effectors to plant COP9 could be coincidental rather than a deliberate attack on the immune system. On the other hand, growing evidence suggests that COP9 function is closely linked to the plant defense mechanism network, and it may have become a prime target for pathogens seeking to manipulate plant immunity [20,61]. The biological importance of COP9 is further emphasized by its homozygous lethality, underscoring its indispensable functions in both plant development and immunity.

## 4. COP9 Regulates Hormone-Mediated Resistance Signaling Pathways

**Figure 3:**
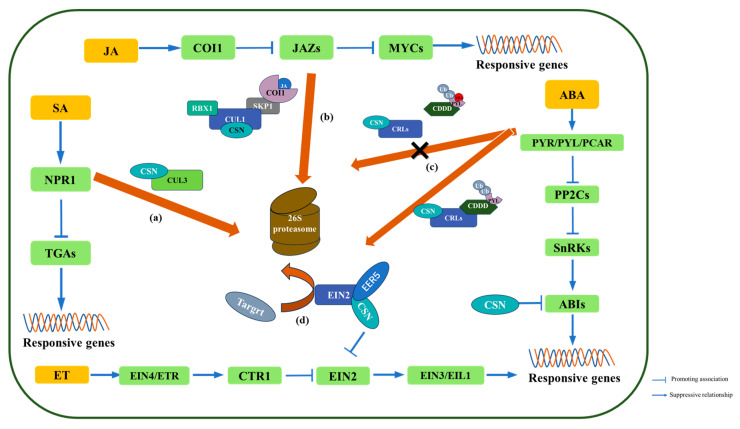
CSN regulates plant hormone signal transduction. (**a**) CSN promotes the degradation of NPR1 by interacting with CUL3. (**b**) Upon sensing JA signals, COI1 forms the SCF^COI1^ complex and degrades JAZ proteins. CSN binds to this complex and facilitates JAZ degradation. (**c**) After the PYL receptor binds ABA, the CDDD module dissociates from CRLs, preventing PYL degradation. CSN is involved in CRL-mediated degradation of PYL. In addition, CSN interacts with EIN2 and influences its function, though the underlying molecular mechanism remains unknown. (**d**) Both EER5 and CSN interact with the C-terminal sequence of EIN2, modulating EIN2’s ability to degrade or modify target proteins.

Phytohormones function as pivotal signaling molecules that mediate plant defense responses against pathogen invasion. Plants employ multiple hormonal signals to respond to biotic stress, with SA, JA, ET, and ABA emerging as key regulators that modulate defense mechanisms in coordination against various pathogens [62]. Notably, recent studies have revealed that the COP9 signalosome serves as a regulatory hub in these phytohormone-mediated defense pathways (Figure 3) [63].

### 4.1. Salicylic Acid

SA is a pivotal phytohormone that orchestrates plant immune responses. Upon pathogen detection, plants rapidly induce SA biosynthesis, resulting in significant SA accumulation during both PTI and ETI. In addition to mediating local defense responses, SA serves a critical role in SAR, enabling whole-plant immune activation [64].

The SA signaling cascade involves dynamic interactions between its receptors NPR1, NPR3, and NPR4, which subsequently activate the transcription factors TGA2/5/6 to induce expression of SA-responsive defense genes [65]. Emerging research highlights the regulatory role of the COP9 signalosome in SA-mediated immunity. For instance, the COP9 complex has been shown to modulate grapevine resistance to powdery mildew through the SA pathway [29]. In wheat, *TaCSN5* negatively regulates broad-spectrum resistance against *Puccinia striiformis* f. sp. *tritici* (*Pst*) by suppressing SA signaling; overexpression lines exhibit enhanced susceptibility accompanied by reduced SA accumulation and downregulation of defense-related genes (*AtPR1* and *TaNPR1*), while RNAi lines show increased resistance [66]. Recent mechanistic studies in rice demonstrate that OsCSN5 maintains immune homeostasis in healthy plants by stabilizing OsCUL3a to promote OsNPR1 degradation. During *Magnaporthe oryzae* infection, pathogen-induced OsPUB45 accumulates and targets OsCSN5 for degradation, leading to decreased OsCUL3a levels and subsequent OsNPR1 accumulation, thereby activating SA-dependent defense responses [26]. This exemplifies how CSN5-mediated stability control of a key ubiquitin ligase component (OsCUL3a) directly regulates the amplitude of SA signaling, positioning CSN at the nexus of hormone homeostasis and immunity.

### 4.2. Jasmonate

The currently known JA signal transduction pathway is mainly composed of the COI1-JA receptor, JAZ (Jasmonate-ZIM) repressors of certain transcription factors [67], and the MYC transcription factor family, which are crucial for JA signaling [68]. In plants, basal levels of JA maintain JAZ proteins in an active state, where they bind to and suppress transcription factors like the MYC2 transcription factor family, thereby repressing JA-responsive genes. However, upon activation of the JA signaling pathway, the COI1 protein senses JA signaling and assembles into the SCF^COI1^ E3 ubiquitin ligase complex, which targets JAZ proteins for proteasomal degradation [69]. This degradation releases MYC and other transcription factors, enabling the expression of JA-responsive genes and amplifying JA biosynthesis, thereby reinforcing JA signaling outputs [69]. CSN plays a critical regulatory role in this process by interacting with the SCF^COI1^ complex and modulating the ubiquitination activity of CRLs [23]. Functioning as a negative feedback regulator, CSN fine-tunes JA signaling and influences plant disease resistance. For instance, *Arabidopsis csn* mutants exhibit hypersensitivity to JA and dysregulated expression of the pathogenesis-related gene *PR1* [70]. Similarly, virus-induced silencing of *AtCSN5* compromises resistance to the necrotrophic fungus *Botrytis cinerea* and reduces endogenous JA levels [71]. Pathogens have evolved mechanisms to exploit this regulatory network. The BYSMV, for example, enhances CSN-mediated suppression of JA signaling to facilitate infection [56]. *Geminiviruses* also manipulate the JA pathway by interfering with COP9 function, thereby disrupting downstream SCF-dependent JA responses [55]. Conversely, in tomato, CSN4 forms a regulatory module with the E3 ubiquitin ligase RING1 to positively regulate JA-dependent defense responses [72]. The opposing effects of different CSN subunits (e.g., general suppression by CSN5 versus positive regulation by CSN4 in specific contexts) hint at a potential subunit specialization in hormone regulation, a theme that warrants further exploration (see Future Perspectives and Experimental Roadmap).

### 4.3. Abscisic Acid

ABA plays a fundamental role in regulating plant developmental processes and mediating environmental stress responses [73,74]. Studies suggest that ABA can either suppress or induce plant defense responses, depending on the type or lifestyle of the pathogen. During viral infection, ABA may contribute to antiviral defense by inducing callose deposition [75]. Upon perception of biotic stress, plants synthesize ABA, which is recognized by the PYR/PYL/RCAR family of receptor proteins [76]. The binding of ABA to these receptors inhibits the phosphatase activity of PP2Cs, thereby relieving their suppression of SnRK2 kinases. Activated SnRK2s, through autophosphorylation, can phosphorylate and activate downstream transcription factors such as ABF/AREB and ABI5, triggering the expression of immune-responsive genes and associated physiological responses [77].

Recent research shows that ABA signaling induces disassembly of the CRL4-CDDD (COP10-DDB1-DET1-DDA1)-CSN complex, primarily through dynamic reorganization of DDA1-CSN interactions [78]. This regulatory switch protects ABA receptors such as PYL8 from ubiquitin proteasome mediated degradation, thereby sustaining ABA signaling during immune challenges. Notably, this protective mechanism can be abolished by CSN5 inhibition, revealing how plants utilize CSN-dependent protein stabilization to amplify hormone signaling under stress conditions. This autoregulatory circuit underscores the COP9 signalosome’s function in orchestrating ABA receptor homeostasis to fine-tune the balance between plant immunity and stress adaptation [79].

Using covalent chemical capture coupled with HPLC–MS/MS analysis, researchers identified several proteins that interact with the ABA receptor PYL5, among them being CSN1, the first subunit of CSN [80]. This finding indicates that CSN may modulate ABA signaling. Additionally, CSN1 represses the effect of ABI5 on seed germination during ABA signaling, but whether it also influences plant immunity remains to be determined [81]. The interaction between PYL5 and CSN1 [72], coupled with CSN’s role in ABA receptor stability [71], solidifies CSN’s role as a key modulator of ABA signaling during stress responses.

### 4.4. Ethylene

Ethylene biosynthesis is strongly upregulated during both PTI and ETI, where it plays a pivotal role in plant immunity. The EIN2-EIN3 signaling module serves as the core regulatory hub in this process; EIN2 not only transcriptionally activates EIN3 but also post-translationally stabilizes it by blocking its ubiquitination and subsequent degradation by the F-box proteins EBF1 and EBF2. This dual regulation amplifies PTI responses by enhancing the expression of defense-related genes [82,83,84]. Fine-tuning of ethylene signaling is achieved through CSN, which interacts with EER5, a PAM domain-containing adaptor protein. EER5 physically links EIN2 and CSN, enabling CSN-mediated degradation of transcriptional repressors downstream of EIN2 (independent of EIN3). This mechanism ensures proper feedback regulation of the ethylene response. Notably, the eer5-1 mutant, which exhibits impaired CSN function, displays hyperactivated ethylene responses, underscoring the critical role of controlled protein turnover in resetting the pathway [85]. This illustrates how CSN integrates with specific adapter proteins (EER5) to provide feedback regulation, ensuring ethylene signaling is potent yet transient, which is crucial for an effective immune response without detrimental side effects.

## 5. COP9 and Immune-Related Secondary Metabolites

Phenylpropanoid phenolic derivatives constitute a major class of secondary metabolites that are essential for plant defense against biotic and abiotic stresses [86]. CSN is linked to the phenylpropanoid pathway; in *Arabidopsis csn5a* mutants, the activity of a trimeric complex involved in both phenylpropanoid derivative synthesis and trichome development is enhanced. Metabolic profiling of *csn5a* seeds further reveals significant accumulations of phenylpropanoids, carotenoids, and a zeatin glycoside [87]. These genetic perturbations lead to substantial reprogramming of secondary metabolism, particularly affecting carotenoid and anthocyanin biosynthesis. Anthocyanins, a prominent subclass of phenylpropanoid-derived flavonoids, function as potent antioxidants and participate in ROS-mediated signaling [88]. Recent studies highlight the context-dependent role of CSN in modulating anthocyanin accumulation across plant species. In tomato, CSN suppresses anthocyanin biosynthesis by facilitating the ubiquitin-mediated degradation of SlBBX20, a key transcriptional activator of the pathway [89]. In contrast, studies on soybean revealed an opposing regulatory mechanism: CSN promotes anthocyanin synthesis under low-phosphorus stress. Overexpression of *GmCSN5A/GmCSN5B* in *Arabidopsis* induces anthocyanin production in shoots, particularly under low-phosphorus conditions, concomitant with the upregulation of core biosynthetic genes (*AtPAL*, *AtCHS*, *AtF3H*, *AtDFR*, and *AtANS*) [90]. These findings demonstrate that CSN-mediated regulation of anthocyanins is species- and condition-specific, reflecting the complex interplay between environmental cues and post-translational control of metabolic pathways. Antimicrobial peptides (AMPs) are small polypeptides, typically comprising fewer than 100 amino acids, that are ubiquitously expressed across various plant tissues. These molecules play a pivotal role in plant innate immunity, serving as a first line of defense against pathogenic invaders. AMPs exhibit dual functionality; they directly neutralize pathogens through antimicrobial activity and indirectly modulate plant immune responses by engaging key signaling pathways, including the MAPK cascade, ROS production, and phytohormone-mediated signaling networks [91]. Emerging research implicates CSN in the regulatory network governing AMP activity. Yeast two-hybrid assays have revealed physical interactions between CSN and two AMP-associated proteins, namely T7F6, a serine endopeptidase inhibitor hypothesized to confer resistance by inhibiting pathogen-derived proteases, and P18C1, a putative defense-related protein whose biological function remains to be fully elucidated [92]. These observations suggest that CSN may post-translationally regulate AMP stability or function through ubiquitin-dependent degradation or by fine-tuning immune-related signaling cascades. However, the mechanistic details underlying CSN-mediated AMP regulation remain incompletely understood, and they include (1) whether specific CSN subunits are preferentially involved in AMP modulation, (2) how pathogen effector proteins might exploit or disrupt this regulatory axis, and (3) the broader physiological relevance of CSN–AMP interactions in plants. Further molecular and genetic studies will be essential to unravel these complexities.

## 6. COP9 Regulation of ROS in Plant Immunity

**Figure 4:**
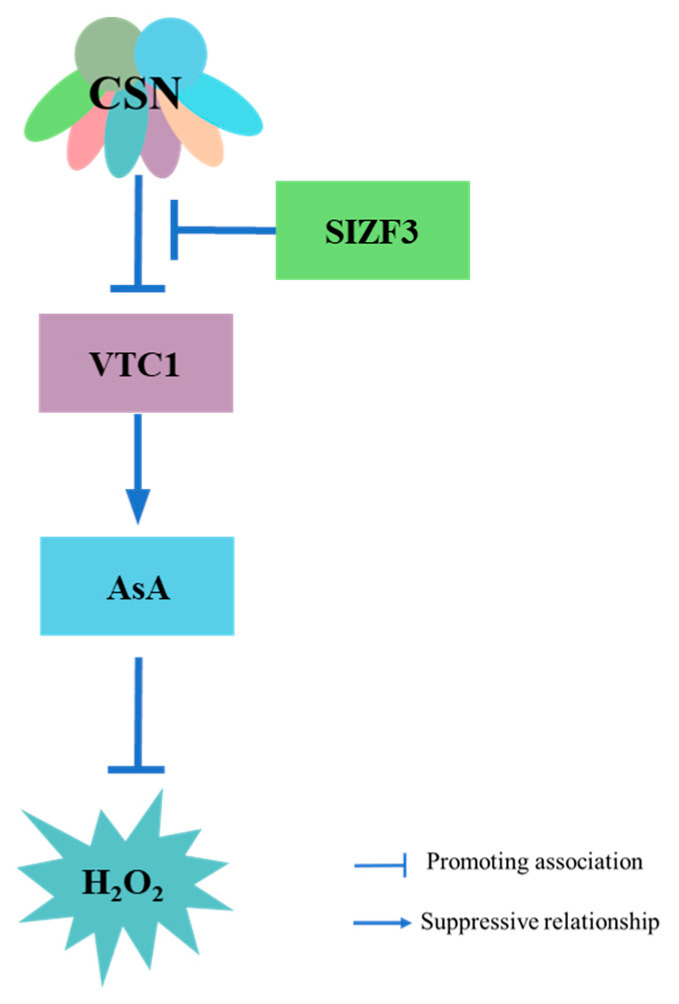
CSN indirectly promotes ROS response by regulating ascorbic acid biosynthesis. CSN promotes the degradation of VTC1, inhibits AsA accumulation, and leads to an increase in reactive oxygen species levels. SIZF3 competes with VTC1 for binding, thereby influencing CSN-mediated degradation of VTC1.

ROS play a dual role in plant defense, serving as signaling molecules at low concentrations to activate immune responses such as PTI and ETI while inducing oxidative damage at elevated levels [93]. The rapid ROS burst following PAMP recognition is crucial for direct pathogen inhibition and the initiation of SAR [94,95]. Ascorbic acid (AsA) is a major antioxidant that neutralizes ROS. The COP9 signalosome fine-tunes ROS homeostasis primarily through the regulation of AsA biosynthesis (Figure 4). Mechanistically, COP9 interacts with VTC1, the rate-limiting enzyme in AsA production, and promotes its degradation via the 26S proteasome pathway. This process suppresses AsA accumulation, leading to elevated ROS levels [96,97,98]. This regulatory axis is further fine-tuned by zinc finger proteins such as SIZF3, which competitively bind to VTC1, disrupting COP9-VTC1 interactions and thereby enhancing AsA synthesis [99]. Although COP9’s role in ROS modulation was first identified under abiotic stress, emerging evidence underscores its significance in biotic stress responses. For instance, rice COP9 mutants display amplified ROS bursts upon chitin treatment and exhibit enhanced resistance to pathogens, consistent with its established function in AsA-dependent ROS regulation [26]. Nevertheless, key questions remain unresolved, including whether the COP9-VTC1 regulatory mechanism is conserved during infection, how tissue-specific variations influence this pathway, and whether pathogen effectors directly interfere with COP9 activity. Additionally, potential crosstalk with other defense signaling networks warrants further investigation.

## 7. COP9 Mediate Regulation of R Gene Homeostasis and Function

NLR proteins serve as central executors of plant immunity, triggering ETI and hypersensitive response (HR) upon pathogen effector recognition, thereby restricting pathogen spread. Maintaining R gene homeostasis is essential for plants; they must launch a swift defense upon a pathogen attack yet keep the HR in check during normal growth to prevent developmental costs. The RAR1-SGT1-HSP90 chaperone complex plays a pivotal role in this process; its CHORD domain interacts with SGT1 to ensure proper NLR protein folding and functional stability. This complex regulates both CC-NB-LRR (CNL)- and TIR-NB-LRR (TNL)-type NLR proteins [100,101,102]. Emerging evidence indicates that CSN negatively modulates plant disease resistance by either directly binding SGT1 or interfering with its interaction with the Skp1-Cullin-F-box (SCF) ubiquitin ligase complex. Genetic studies demonstrate that CSN silencing compromises tobacco mosaic virus (TMV) resistance, underscoring its essential role in R gene-mediated immunity [103,104,105]. Intriguingly, SGT1 exhibits dual functionality; beyond its chaperone role in immunity, it regulates cell cycle progression (G1/S and G2/M transitions) and suppresses SKP1 in yeast SCF complexes. However, whether these cell cycle-related functions intersect with COP9-mediated ubiquitination pathways remains an open question [106].

The COP9 signalosome exerts broad-spectrum resistance effects through its regulation of the RAR1-SGT1-HSP90 complex, a mechanism conserved across NLR structural types [107]. Enhanced disease resistance and abnormal NLR protein accumulation have been observed in cop9 mutants across plant species [28,104], yet key questions remain. Does COP9 regulate NLR stability via the ubiquitin-proteasome pathway? Do pathogen effectors target this regulatory node? Furthermore, the potential crosstalk between SGT1-mediated cell cycle and immune signaling warrants exploration. Deciphering the COP9-R gene regulatory network will not only advance understanding of plant immune homeostasis but also inform novel NLR-based strategies for crop disease resistance breeding. Future research should focus on elucidating COP9’s interaction specificity with different NLR classes and its regulatory patterns in plant-pathogen coevolution.

## 8. Concluding Remarks and Future Perspective

### 8.1. An Integrated Model for CSN in Plant Immunity: The Central Regulatory Hub

**Figure 5:**
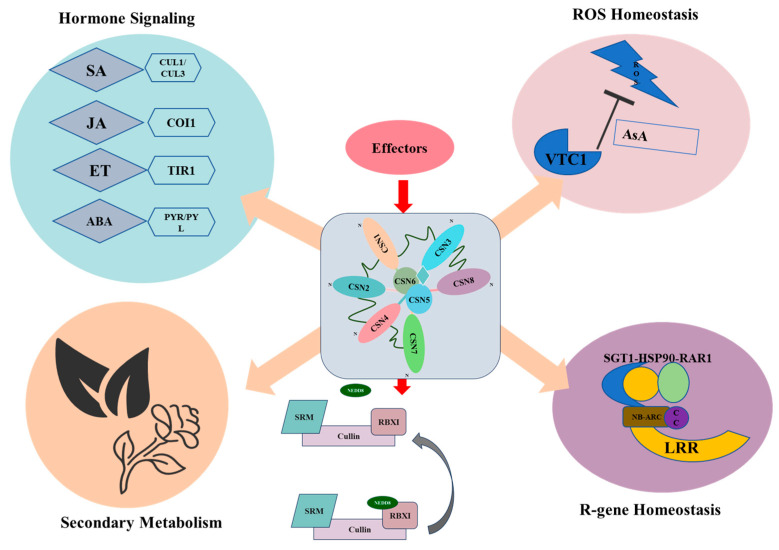
Proposed integrative model of CSN as a central hub in plant immunity. The CSN complex, through its deneddylation activity (CSN5/6) and scaffold, regulates multiple immune pathways by controlling the stability and activity of key components. These include CRLs involved in hormone signaling (SA, JA, ABA, and ET), the VTC1 enzyme in ascorbate (AsA) and ROS homeostasis, factors influencing SM and AMPs, and the SGT1-HSP90 chaperone system governing NLR receptor stability. PAMPs or effectors may dynamically influence CSN activity. Conversely, numerous pathogen effectors directly target CSN, particularly the CSN5 subunit, to disrupt this regulatory hub and suppress immunity.

Current research demonstrates that the COP9 signalosome serves as a regulatory node integrating multiple plant immune pathways, including effector recognition, ROS homeostasis, defense hormone signaling, R protein function, and antimicrobial metabolite production. While its canonical role in ubiquitin-mediated protein degradation has been well established, emerging evidence suggests COP9 may also participate in phosphorylation-dependent signaling and transcriptional regulation. However, a major gap remains in understanding how CSN coordinates these diverse functions during pathogen infection. We propose a unified, albeit speculative, model (Figure 5) where CSN acts as a central processing hub.

In this model, the intact CSN complex, through its deneddylation activity (primarily via CSN5/6) and scaffolding functions (other subunits), exerts broad yet precise control over key immune components: CRLs for hormone signaling (SA, JA, ABA, and ET), the VTC1-AsA axis for ROS homeostasis, transcription factors (e.g., SlBBX20) and E3 ligases for secondary metabolism and the SGT1-HSP90 chaperone system for NLR stability.

This regulation is dynamic and context-dependent. Pathogen perception may trigger signaling events (e.g., Ca^2+^ flux or MAPK activation) that modulate CSN activity, composition, or subcellular localization, potentially shifting its function to prioritize defense over growth.

The model highlights why CSN is a prime target for pathogens. Effector proteins (e.g., PcSnel4B, P5-1, and C2) frequently target CSN5, as inhibiting this catalytic core represents an efficient strategy to disrupt the entire hub and suppress multiple immune outputs simultaneously. This model provides a framework for future experiments to test the coordination and prioritization mechanisms within the CSN immune network.

### 8.2. Functional Specialization and Coordination of COP9 Subunits

While the COP9 signalosome functions as an integrated complex in plant immunity, emerging evidence reveals distinct functional specializations among its subunits that warrant systematic investigation. The canonical eight subunits exhibit both conserved and specialized roles, with CSN5’s JAMM domain mediating critical deneddylation activity while CSN1 and CSN2 maintain structural integrity and other subunits (CSN3,4,6–8) participate in substrate recognition. Notably, mammalian studies demonstrate antagonistic functions between CSN5 (proliferation promoter) and CSN8 (proliferation suppressor) [108], suggesting similar regulatory complexity may exist in plants. The opposing effects on JA signaling (suppression by CSN5 versus positive regulation by CSN4 in tomato [64]) and the specific targeting of CSN5 by numerous effectors support this notion. Key unresolved questions include the extent of subunit redundancy versus specialization in immune responses, potential tissue-specific expression patterns, and how pathogen effectors might exploit subunit-specific functions. Future research employing subunit-specific mutants, structural analyses, and high-resolution interactome mapping under different immune conditions is crucial to elucidate how individual subunits contribute to the signalosome’s integrated function while maintaining specialized roles.

### 8.3. COP9 as a Central Regulator of the Growth-Defense Trade-Off

The COP9 signalosome plays a fundamental role in promoting plant growth and development, with well-documented functions in diverse processes such as photomorphogenesis, fruit ripening [109], seed germination [25], and tiller formation in monocots. Genetic studies demonstrate that COP9 overexpression enhances growth vigor, while complete loss-of-function mutations typically lead to embryonic lethality [110], highlighting its essential role in basic developmental programs. These observations position COP9 as a master positive regulator of plant growth, likely through its conserved function in modulating the activity of the Cullin-RING E3 ubiquitin ligases that control key developmental signaling pathways.

Current research generally holds that CSN functions as a negative regulator of disease resistance. Genetic evidence consistently demonstrates that silencing or mutating core subunits (particularly CSN5) enhances pathogen resistance, whereas their overexpression suppresses immune responses. This inverse relationship strongly supports the classical growth-defense trade-off paradigm, positioning COP9 as a central regulatory hub that orchestrates resource allocation between these competing biological priorities. This inverse relationship strongly supports the classical growth-defense trade-off paradigm, positioning CSN as a central regulatory hub that orchestrates resource allocation between these competing biological priorities. Three fundamental questions emerge from these observations. (1) What molecular mechanisms enable COP9’s ubiquitination machinery to differentially target growth-promoting versus defense-related substrates? (2) As hinted at in Section 4.2 and Section 8.2, do individual COP9 subunits exhibit functional specialization, with distinct subunits or sub-complexes dedicated to developmental or immune processes? (3) Finally, how do pathogen-associated molecular patterns dynamically modulate COP9 activity to reprogram the growth-defense equilibrium? Addressing these knowledge gaps through tissue-specific genetic approaches, coupled with temporal profiling of COP9 substrate networks, could yield transformative insights. Such investigations may uncover novel biotechnological strategies for developing crop varieties with an optimized balance between growth vigor and disease resistance.

### 8.4. Future Perspectives and Experimental Roadmap

To move beyond correlation and fully unravel the mechanistic underpinnings of CSN’s role in immunity, future studies should employ integrated approaches.

Dynamic Interactomics: Comprehensive interactome mapping (e.g., using TurboID) of CSN complexes under different infection conditions and time points can identify condition-specific partners and substrates.

Functional Dissection: Development and characterization of separation-of-function mutants (e.g., catalytic dead CSN5 or interaction domain mutants of other subunits) can distinguish between deneddylation-dependent and independent activities of CSN in immunity.

Real-Time Monitoring: Employing live-cell imaging and biosensors can help track CSN complex dynamics, localization, and activity in real-time during immune responses.

Structural Insights: Structural biology approaches (e.g., Cryo-EM or X-ray crystallography) can help determine how CSN interacts with different CRLs, immune components, and pathogen effectors, revealing the basis for substrate recognition and manipulation.

Toward Application: Exploring the potential of engineering CSN subunits or their interactors can help fine-tune the growth-defense balance, aiming to develop crop varieties with enhanced resilience without significant yield penalties.

## Data Availability

No new data were created or analyzed in this study. Data sharing is not applicable to this article.

## Abbreviations

The following abbreviations are used in this manuscript:

ABA	Abscisic acid
AMPs	Antimicrobial peptides
AsA	Ascorbic acid
BYSMV	Barley yellow striate mosaic virus
COI1	Coronatine Insensitive 1
CNL	CC-NB-LRR
CRLs	Cullin-RING E3 ubiquitin ligases
CSN	COP9 signalosome
CSNAP	COP9 signalosome-associated acidic protein
ET	Ethylene
ETI	Effector-triggered immunity
HR	Hypersensitive response
JA	Jasmonate
JAZ	Jasmonate-ZIM
LC-MS	Liquid chromatography–mass spectrometry
MAPK	Mitogen-activated protein kinase
NLR	Nucleotide-binding leucine-rich repeat
NPR1	Nonexpressor Of PR genes 1
PAMPs	Pathogen-associated molecular patterns
PCD	Programmed cell death
PCI	Proteasome-COP9-eIF3
PR	Pathogenesis-related
PRRs	Pattern recognition receptors
PTI	Pattern-triggered immunity
RBSDV	Rice black -streaked dwarf virus
ROS	Reactive oxygen species
SA	Salicylic acid
SAR	Systemic acquired resistance
SLY1	Sleepy 1
SM	Secondary metabolism
TIR1	Transport inhibitor response 1
TNL	TIR-NB-LRR
UFO	Unusual flower organs
UPS	Ubiquitin-proteasome
